# Wastewater-Based Epidemiology and Long-Read Sequencing to Identify Enterovirus Circulation in Three Municipalities in Maricopa County, Arizona, Southwest United States between June and October 2020

**DOI:** 10.3390/v13091803

**Published:** 2021-09-10

**Authors:** Temitope O. C. Faleye, Devin A. Bowes, Erin M. Driver, Sangeet Adhikari, Deborah Adams, Arvind Varsani, Rolf U. Halden, Matthew Scotch

**Affiliations:** 1 Biodesign Center for Environmental Health Engineering, Biodesign Institute, Arizona State University, Tempe, AZ 85287, USA; tfaleye@asu.edu (T.O.C.F.); dbowes@asu.edu (D.A.B.); Erin.Driver@asu.edu (E.M.D.); sadhik14@asu.edu (S.A.); rhalden@asu.edu (R.U.H.); 2 School of Sustainable Engineering and the Built Environment, Arizona State University, Tempe, AZ 85287, USA; 3 Biodesign Center for Personalized Diagnostics, Biodesign Institute, Arizona State University, Tempe, AZ 85287, USA; dadams14@asu.edu; 4 Biodesign Center for Fundamental and Applied Microbiomics, School of Life Sciences, Center for Evolution and Medicine, Arizona State University, Tempe, AZ 85287, USA; arvind.varsani@asu.edu; 5 OneWaterOneHealth, Nonprofit Project of the Arizona State University Foundation, Tempe, AZ 85287, USA; 6 College of Health Solutions, Arizona State University, Phoenix, AZ 85004, USA

**Keywords:** wastewater-based epidemiological monitoring, Enterovirus C, human, high-throughput nucleotide sequencing, Arizona, environmental monitoring

## Abstract

We used wastewater-based epidemiology and amplicon-based long-read high-throughput sequencing for surveillance of enteroviruses (EVs) in Maricopa County, Arizona, Southwest United States. We collected 48 samples from 13 sites in three municipalities between 18 June and 1 October 2020, and filtered (175 mL each; 0.45 µm pore size) and extracted RNA from the filter-trapped solids. The RNA was converted to cDNA and processed through two workflows (Sanger sequencing (SSW) and long-read Illumina sequencing (LRISW)) each including a nested polymerase chain reaction (nPCR) assay. We subjected the ~350 bp amplicon from SSW to Sanger sequencing and the ~1900–2400 bp amplicon from LRISW to Illumina sequencing. We identified EV contigs from 11 of the 13 sites and 41.67% (20/48) of screened samples. Using the LRISW, we detected nine EV genotypes from three species (Enterovirus A (CVA4, EV-A76, EV-A90), Enterovirus B (E14) and Enterovirus C (CVA1, CVA11, CVA13, CVA19 and CVA24)) with Enterovirus C representing approximately 90% of the variants. However, the SSW only detected the five Enterovirus C types. Similarity and phylogenetic analysis showed that multiple Enterovirus C lineages were circulating, co-infecting and recombining in the population during the season despite the SARS-CoV-2 pandemic and the non-pharmaceutical public health measures taken to curb transmission.

## 1. Introduction

Enteroviruses (EVs) are non-enveloped, positive-sense, single-stranded RNA viruses that belong to the genus Enterovirus, family *Picornaviridae*, order *Picornavirales*. Within the genus, there are over 300 serologically distinct genotypes categorized into 15 species (Enterovirus A to L and Rhinovirus A–C) [1]. The polioviruses (serotypes 1, 2 and 3) are the best studied members of the genus Enterovirus and are members of species Enterovirus C alongside 22 other serotypes, including coxsackievirus (CV)A1, CVA11, CVA13, CVA19 and CVA24. As a result of establishing a correlation between EV serotypes and their VP1 gene sequence [2], EV typing by serological assays has been replaced by VP1 gene, complete capsid, or whole-genome sequence analysis [3].

Annually in the USA, EVs cause approximately 15 million infections and tens of thousands of hospitalizations [4] with a wide range of clinical manifestations that range from mild (e.g., runny nose and fever) to severe presentations such as intra-uterine infection with fetal death and acute flaccid myelitis (AFM) [5,6].

Recently, wastewater-based epidemiology (WBE) has been employed to monitor viruses in the population as an alternative or complementary approach to case-based surveillance (CBS). The WBE approach is especially important for EV surveillance and molecular epidemiology, considering only 0.5–1% of infections show clinical manifestations, but all EV-infected individuals shed virus in high titers (~10^8^ virus particles per gram) in feces [7]. Previous studies have used WBE for detecting EV presence and, more importantly, describing EV dynamics in populations [8,9,10,11,12,13].

The current SARS-CoV-2 pandemic has overwhelmed pathogen surveillance globally, resulting in reduced surveillance of other pathogens [14]. It has, however, been suggested that the observed decrease in detection of other viruses (including those transmitted via non-respiratory routes such as EVs) might not be the result of reduced surveillance. Rather, it is thought that non-pharmaceutical measures taken to mitigate SARS-CoV-2 transmission have also impacted pathogens transmitted via the fecal–oral route such as EVs [15,16].

In this study, we use WBE for EV surveillance (as a prototype for viruses transmitted via the fecal–oral route) over a 15 week period during the traditional peak of the EV transmission season [17] in three municipalities in Maricopa County, Arizona, Southwest, USA. We show that even at the height of the SARS-CoV-2 pandemic, EV circulation was ongoing in this population. Our results show the importance of WBE for virus surveillance at the population scale, especially in conditions where clinical or case-based pathogen diagnostics is inadequate or overwhelmed.

## 2. Materials and Methods

In this study, we collected forty-eight 24 h composite wastewater (WW) samples by time or flow-weighted automated samplers over the course of 15 weeks (18 June–1 October 2020) from 13 sites in three municipalities in Maricopa County, Arizona, Southwest USA as part of an ongoing wastewater-based epidemiology (WBE) virus monitoring study. These samples are from municipalities that have a total population of over 700,000 people [18] and were collected on days 0, 28, 70 and 105 from each of the 13 sites. Four WW samples were collected from each site except in four cases where one sample was not collected for logistical reasons. This included site 13-day 0, sites 3 and 11-day 28, and site 7-day 70. We transported the samples on ice to our laboratory at Biodesign Institute, Arizona State University, Tempe, AZ, USA, where they were processed. Precisely, 175 mL of WW was first clarified by filtration through a 450 nm polyethersulfone filter using a Nalgene Rapid-Flow single-use, disposable membrane vacuum filtration unit (Thermo Fisher Scientific, Waltham, MA, USA).

We collected all 450 nm membrane filtration cups used for clarifying the WW samples and retrieved the individual filters. We subsequently added the filters to a 50 mL centrifuge tube containing 4 mL of sterile PCR-grade water and 1 g of plastic beads (Horizon group, Warren, NJ, USA). The tubes were vortexed for 5 min (or until filters were clean) using Vortex Genie (Scientific Industries, Bohemia, NY, USA) at maximum speed. Afterwards, we removed the filters, centrifuged the extract at 4000 rpm for 20 min, collected the supernatant, and stored it in aliquots at −80 °C for further analysis.

**Figure 1:**
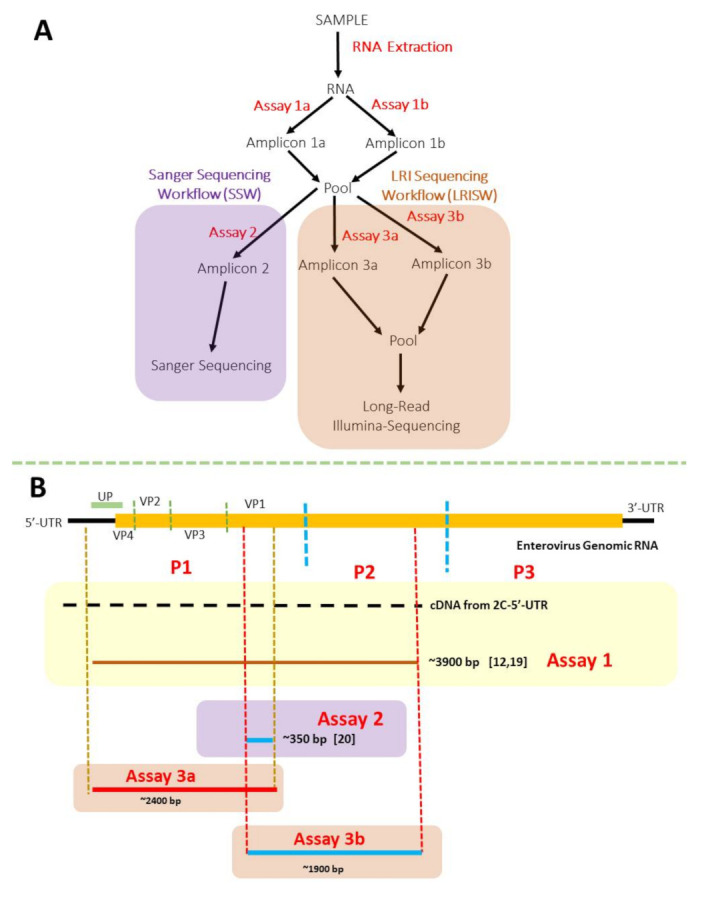
(**A**) Summary of the workflow used in this study. (**B**) The region of enterovirus genomes amplified in each of the assays. Abbreviation: LRI = long-read Illumina.

We used two workflows in this study—the Sanger sequencing workflow (SSW) and the long-read Illumina sequencing workflow (LRISW) (Figure 1A). Particularly, the SSW was used to cost effectively streamline samples that went into the LRISW. Furthermore, both workflows also served as controls for each other as all nucleic acid sequencing reported in this study were done in two independent commercial sequencing facilities. For each of the 48 samples, 280 µL (140 µL × 2) was used for RNA extraction using the QiaAmp RNA extraction kit (Qiagen, Germantown, MD, USA) following manufacturer’s recommendations. The resulting RNA (final volume 80 µL) was then used in the one-step RT-PCR assays described by Majumdar and Martin [12]. We added 5 µL of RNA to a 20 µL mix containing 12.5 µL of Superscript III RT-PCR buffer (Invitrogen), 1 µL (10 µM) each of both forward and reverse primers [12], 0.5 µL of RT/HiFi enzyme and 5 uL of PCR-grade water. The mixture was amplified in a thermal cycler (BioRad, Hercules, CA, USA) with the following cycling conditions: 50 °C for 30 min followed by 94 °C for 2 min and 42 cycles of 94 °C for 15 s, 55 °C for 30 s, and 68 °C for 8 min. This was followed by 68 °C for 5 min and 4 °C until stopped. We pooled the product of the assays (per sample) and used them as template for assays 2 (SSW), 3a and 3b (LRISW) (Table 1, Figure 1A).

We used GoTaq green (Promega Corporation, Madison, WI, USA) and Phusion High-Fidelity (New England Biolabs Inc., Ipswich, MA, USA) PCR kits for the SSW and LRISW, respectively (Figure 1A). For both workflows, we added 2 µL of the pooled product (from assays 1a and 1b) to a 23 µL mix containing 12.5 µL of PCR master mix, 0.25 µL (100 µM) of each of forward and reverse primers and 10 µL of PCR-grade water. Amplification was performed in a thermal cycler (BioRad, Hercules, CA, USA) (see Table 1 for cycling conditions). We specified an initial denaturation at 94 °C for 2 min followed by 94 °C for 30 s per cycle. All three assays also included a final stage of extension temperature for 10 min followed by 4 °C until stopped. We resolved amplicons <1000 bp and those >1000 bp (Figure 1B) on 2% and 1% agarose gels, respectively. All gels were stained with GelRed (Biotium, Fremont, CA, USA) and viewed using a GelDoc imager (Bio-Rad, Hercules, CA, USA).

We purified the ~350 bp amplicons (assay 2) generated in the SSW using the Qiagen PCR purification kit (Qiagen, Germantown, MD, USA). The amplicons were Sanger sequenced at the Genomics Core, Biodesign Institute, Arizona State University, Tempe, AZ, USA, using both primers AN89 and AN88. The amplicons generated in the LRISW (assay 3a ~2400 bp and 3b ~1900 bp), were pooled per sample and purified using the Qiagen PCR purification kit (Qiagen, Germantown, MD, USA). Afterwards, the amplicons were indexed, purified, normalized, and pooled using the Loop Genomics (San Jose, CA, USA) kit as recommended by the manufacturer. The pooled sample was shipped to Loop Genomics where intra-molecular tagging, library preparation, paired-end (2 × 150) Illumina sequencing (on the HiSeq 4000 Illumina sequencer) and long-read assembly were done using SPAdes [21]. We typed all contigs using the enterovirus genotyping tool [22].

It is important to mention that the Loop Genomics long-read technology leans on the random intra-polynucleotide incorporation of unique barcodes added to the ends of each polynucleotide in an amplicon during indexing. During demultiplexing (i.e., post-Illumina sequencing), the small reads are first sorted into samples and subsequently into intra-sample clusters based on the intra-polynucleotide unique barcodes. Each cluster is then de novo assembled into long reads [23].

For each EV genotype identified, we used ClustalW in MEGAX [24] for multiple sequence alignment. Subsequently, duplicate contigs were removed using the ‘find duplicates’ function in Geneious Prime [25]. We used each deduplicated alignment for similarity and phylogenetic analysis using MEGAX [24]. Similarity analysis was done using the compute pairwise distance tool in MEGAX [24]. For phylogenetic analysis, both neighbor joining (NJ) and maximum likelihood (ML) trees were made using the Kimura-2-parameter and GTR model, respectively, and a 1000 bootstrap replicates in MEGAX [24]. Phylogenetic trees encompassing all Enterovirus C species recovered (>300 sequences) were first made using the NJ algorithm, subtrees with topologies not clustered by type were extracted and reanalyzed using both NJ and ML trees as described above. All EV type-specific alignments were made using the ML algorithm.

For the EV species with the most diversity, to our deduplicated alignment, we added sequences recovered from GenBank [26] using a BLASTn [27] search of the database using our sequence of interest as query. The updated alignment was used to infer ML trees as described above. We used the following nomenclature for variants described in this study in the phylogenetic trees; ST4-d0-MOLECULE-972 means Site 4-day0-contig-972.

We deposited all the EV sequences generated in this study in NCBI GenBank [26]. Contigs generated by Sanger sequencing are under the accession numbers MW817168-MW817183. Contigs generated by long-read Illumina sequencing are under the accession numbers MZ561707-MZ562246 and MZ579712-MZ580089.

## 3. Results

### 3.1. Nested PCR (SSW and LRISW)

#### 3.1.1. SSW

Precisely, 58.33% (7/12), 36.36% (4/11), 50% (6/12) and 61.54% (8/13) of the sites sampled at the four (4) sampling time points (days 0, 28, 70 and 105, respectively) resulted in the detection of amplicons suggestive of EV presence in the samples (Table 2). Additionally, while the expected amplicon size suggestive of EV presence was detected at all thirteen sampling sites, only 53.85% (7/13) of the sites yielded the signal at least 50% of the time. In all, 52.08% (25/48) of the samples collected had amplicon size suggestive of EV presence in the samples.

#### 3.1.2. LRISW

It is important to emphasize that only the 25 samples that yielded amplicons (~350 bp, as viewed through an agarose gel imager) suggestive of EV presence in the SSW were subjected to the LRISW (Table 2 and Table S1). Twenty (80%) of the 25 samples were positive for the LRISW and subjected to long-read Illumina sequencing (Table 2). The remaining five (5) samples were negative for both PCR assays (3a and 3b in Figure 1A) in the LRISW.

### 3.2. Sequencing and EV Type Determination

#### 3.2.1. SSW and EV Type Determination

**Figure 2:**
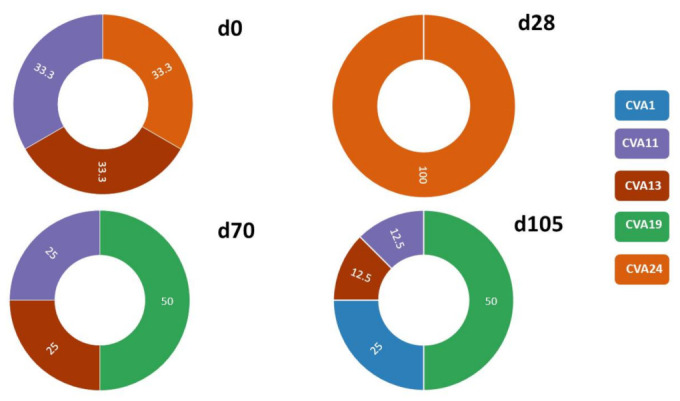
Relative abundance of enterovirus variants recovered and unambiguously identified on the four (0, 28, 70 and 105) sampling days using the SSW. Numbers in chart are percentages reflecting the number of sites where the different EV types were detected relative to the number of sites where EVs were detected and unambiguously typed on any specific sampling day. Please note that d0, d28, d70 and d105 mean EV types detected in samples collected on days 0, 28, 70 and 105, respectively. Additionally, note that 100% in d28 refers to the single CVA24 detected on that day (please see Table 2 and Table S1).

Of the 25 samples with the expected amplicon size, we unambiguously identified EVs in 16 (64%). The remaining nine (9) samples had multiple peaks in the Sanger sequencing chromatograms suggesting that more than one EV type might be present. The 16 identifiable samples had five EV types, CVA1 (2 variants), CVA11 (3 variants), CVA13 (3 variants), CVA19 (6 variants) and CVA24 (2 variants) (Table 2 and Table S1). It is important to note that all are Enterovirus C species members. Viewed across sampling days, days 0, 28, 70 and 105 yielded three (CVA11, CVA13 and CVA24), one (CVA24), three (CVA11, CVA13 and CVA19) and four (CVA1, CVA11, CVA13 and CVA19) EV types, respectively (Figure 2) using the SSW.

#### 3.2.2. LRISW and EV Type Determination

Precisely, 25,387,209 short-read sequences were generated from the 20 samples subjected to Illumina sequencing. These short reads were assembled into 61,197 long-reads (Table S1), 1243 of them had lengths corresponding to contigs from assays 3a and 3b (Figure 1B) and were confirmed as EV contigs using the enterovirus genotyping tool [22]. We removed duplicate (100% identical) contigs, leaving 918 (393 and 525 from assays 3a and 3b, respectively) unique EV contigs (Table 2). These contigs were recovered from 19 (95%) of the 20 samples. In Table 3, we show the range in number of short reads that mapped to each EV contig in these 19 samples. Coverage per contig ranged from ~236× to 19× for the ~2400 bp (i.e., assay 3a) contigs and 368× to 16× for the ~1900 bp (i.e., assay 3b) contigs (Table 3). The one sample in which we could not detect EV with the expected contig size was from Site-5-day-28 (Table 2).

In all, members of three EV species distributed into nine (EVA (CVA4, EV-A76, EV-A90), EVB (E14) and EVC (CVA1, CVA11, CVA13, CVA19 and CVA24)) EV types were recovered using the LRISW. It is important to note that 93.13% (366/393) and 89.14% (468/525) of the variants recovered from assays 3a and 3b, respectively, are Enterovirus C members (Table 2).

**Figure 3:**
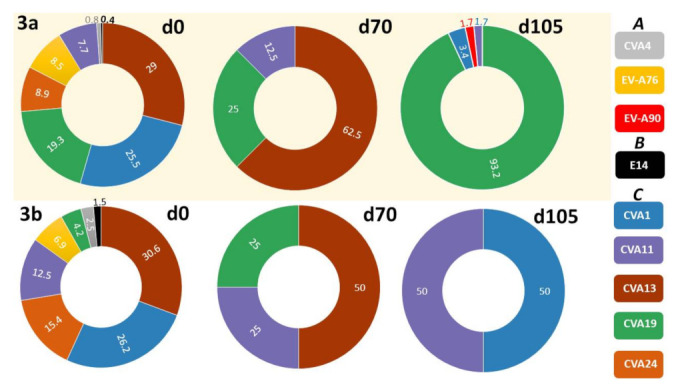
Relative abundance of enterovirus variants recovered on three (3) of the sampling days (0, 70 and 105) using assays 3a and 3b (LRISW). Numbers in chart are percentages reflecting the cumulative number of variants across all sites where the different EV types were detected on any specific sampling day. Note that the image above only displays variants recovered using assays 3a and 3b (LRISW). Particularly, the CVA24 and CVA13 recovered on days 28 and 105, respectively, using assay 2 (SSW) are not shown here. Note that d0, d70 and d105 mean EV types detected in samples collected on days 0, 70 and 105, respectively.

When considering distribution across sampling days, days 0, 28, 70 and 105 yielded eight (EVA (CVA4, EV-A76), EVB (E14) and EVC (CVA1, CVA11, CVA13, CVA19 and CVA24)), zero, three (EVC (CVA11, CVA13 and CVA19)) and four (EVA (EV-A90) and EVC (CVA1, CVA11 and CVA19)) EV types, respectively (Figure 3) using the LRISW.

#### 3.2.3. Comparison of EV Types Identified Using SSW and LRISW

In this study, using the SSW (Figure 1A), we detected five EV types (CVA1 (2 variants), CVA11 (3 variants), CVA13 (3 variants), CVA19 (6 variants) and CVA24 (2 variants)) in 16 samples (Table 2). We also recovered all five EV types using the LRISW. Except for two cases, there was congruence between EV types detected per sample by the SSW and those detected by the LRISW. The two exceptions were in samples site-1-day-105 and site-5-day-28 that yielded CVA13, and CVA24, respectively, by the SSW but EVA90 and nothing by the LRISW (Table 2).

Two samples (site-5-day-0 and site-11-day-105) yielded more EV types by the LRISW than detected using the SSW. In sample site-5-day-0, in addition to CVA24 recovered by both methods, the LRISW also detected the presence of CVA4. In sample site-11-day-105, in addition to CVA1 recovered by both methods, the LRISW also detected the presence of CVA19 (Table 2).

Nine of the amplicons recovered by the SSW yielded multiple peaks (Table 2). Of these nine samples, only four (site-3-day-0, site-4-day-0, site-7-day-0, and site-9-day-0) yielded amplicons by the LRISW. Specifically, using the LRISW, site-3-day-0, site-4-day-0, site-7-day-0 and site-9-day-0 yielded three (EV-A76, E14 and CVA19), four (EV-A76, CVA1, CVA19 and CVA13), five (EV-A76, CVA1, CVA11, CVA13 and CVA19) and two (CVA1 and CVA19) EV types, respectively. The remaining five samples were negative suggesting they might be non-specific amplification products of assay 2 (SSW).

Viewed across sampling days, the LRISW detected all EV types detected by the SSW except CVA24 on day 28 and CVA13 on day 105 (Figure 2, Table 2). On the other hand, the LRISW showed that in addition to the EV types recovered by the SSW, CVA4, EV-A76, E14, CVA1 and CVA19 were present in the population on day 0 and EV-A90 was present on day 105 (Figure 3).

### 3.3. Pairwise Distance and Phylogenetic Analysis

Pairwise distance analysis of all EV types recovered using the LRISW showed divergence ranging from 0.65% to 26.16% and 0.48% to 28.08% using LRISW assays 3a and 3b, respectively (Table S2). When viewed by day of sampling, divergence per EV type in one sample varied from 0.09% to 24.37% and 0.05% to 28.08% using LRISW assays 3a and 3b, respectively (Table S1). It should be noted that assay 3b contigs span VP1-2C genomic region. Hence, the divergence described in Table 2, Tables S1 and S2 cover VP1-2C and does not represent divergence in the P2 (2A-2C) genomic region only.

**Figure 4:**
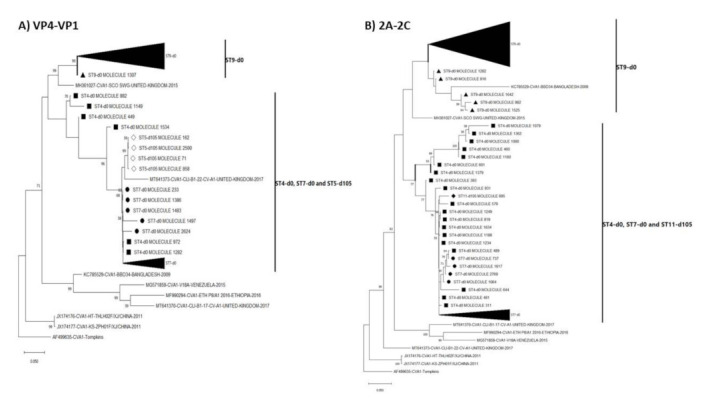
Maximum likelihood phylogenetic tree of CVA1 (**A**) VP4-VP1 [from assay 3a contigs] and (**B**) 2A-2C [from assay 3b contigs] genomic regions. The phylogenetic trees are based on an alignment of CVA1 contigs recovered from assay 3a and 3b (LRISW) in this study and those present in GenBank. The contigs are coded based on the sampling sites and days. Filled triangle, circle, square and diamond represent ST9-d0, ST7-d0, ST4-d0 and ST11-d105, respectively. ST5-d105 is denoted using empty diamond. Bootstrap support is shown if >50. Abbreviation: ST = site. Please note that the region amplified in the SSW is present in the contig used for making the tree in Figure 4A but not in Figure 4B. Specifically, all VP1 portions of amplicon 3b were removed from the contigs used to make the tree in Figure 4B.

We performed phylogenetic analysis for all EV types with >15% divergence (Table S2) and all showed (with strong bootstrap support) that multiple lineages were present and circulating in the population (Figures S1–S3). For the EV type (CVA1) with most divergence (exceeding 20% in contigs recovered using both assays 3a and 3b, Table S2) we included representative sequences from GenBank to the alignment and showed that the topology of both trees remained consistent that in Figure S1 further confirming that multiple lineages were present and circulating in the population (Figure 4).

**Figure 5:**
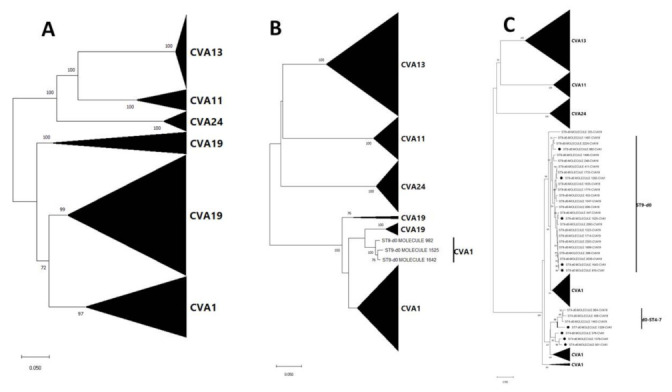
Neighbor joining phylogenetic tree of Enterovirus Cs recovered in this study (**A**) VP4-VP1 [from assay 3a contigs] (**B**) VP1-2C [assay 3b contigs] and (**C**) 2A-2C [from assay 3b contigs] genomic regions. The phylogenetic trees are based on an alignment of Enterovirus C contigs recovered from assay 3a and 3b (LRISW) in this study. In Figure 5C, the CVA1 contigs that do not cluster with other CVA1s are indicated with black circles. Bootstrap support is shown if >50. Abbreviation: ST = site. Collapsed taxa denote all contigs that belong to the same EV type.

**Figure 6:**
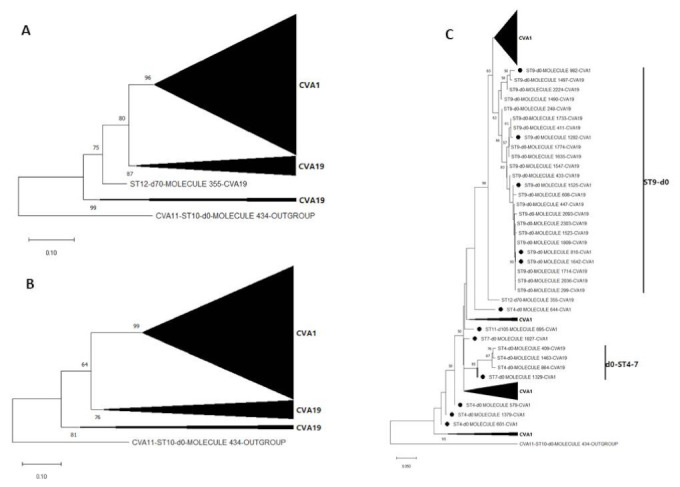
Maximum likelihood phylogenetic tree of CVA1 and CVA19 contigs recovered from assay 3b (LRISW) in this study (**A**) VP1-2C (**B**) VP1 only and (**C**) 2A-2C. In Figure 6C, the CVA1 contigs that do not cluster with other CVA1s are indicated with black circles. Bootstrap support is shown if >50. Abbreviation: ST = site. Collapsed taxa denote all contigs that belong to the same EV type.

NJ phylogenetic trees of the most abundant EV species detected (species C) showed that all assay 3a (VP4-VP1) contigs (LRISW) clustered by type but phylogeny violations were observed for CVA1 and CVA19 in the assay 3b (VP1-2C and 2A-2C) contigs (Figure 5). Particularly, for the VP1-2C tree (Figure 5) CVA1 contigs 982, 1525 and 1642 clustered with CVA19. For these contigs we repeated their identification using EGT and BLASTn and confirmed they were indeed CVA1 (Figure S4). We therefore made NJ and ML phylogenetic trees of CVA1 and CVA19 contigs only, and these showed that the phylogeny violation was only in the 2A-2C genomic region (Figure 6 and Figure S5).

These phylogeny violations fell into two clusters, CVA1 and CVA19 from ST9-d0 and another involving CVA19 from ST4-d0 and CVA1 from ST7-d0 (Figure 5, Figure 6 and Figure S5). Hence, suggesting recombination (as a result of CVA1 and CVA19 co-infection) might be occurring in the 2A-2C (P2 genomic region) of CVA1 and CVA19 variants circulating in the population. However, though the assays were run with enzymes with high fidelity and processivity, for samples from ST9-d0, because variants of both CVA1 and CVA19 genomic regions were in the mix during PCR amplification it is difficult to rule out the possibility that this recombination signal could be due to template switching during the PCR amplification process. However, this is less likely for the signal involving CVA19 from ST4-d0 and CVA1 from ST7-d0 as both were from different samples.

## 4. Discussion

In this study, we set out to determine whether EVs (a prototype of viruses transmitted via the fecal–oral route) were circulating in three municipalities in Maricopa County, AZ, USA over the course of 105 days (18 June to 1 October) in 2020, during the historical annual peak of EV transmission. Our findings show that despite the SARS-CoV-2 pandemic and non-pharmaceutical interventions, EVs were circulating as expected during the season. Particularly, in this study, we showed that at least nine (9) different EV types belonging to three different species (EVA (CVA4, EVA76, EVA90), EVB (E14) and EVC (CVA1, CVA11, CVA13, CVA19 and CVA24)) were present and circulating (Figure 2, Figure 3 and Figure 4 and Figures S1–S3) in the population over the 105 days (15 weeks) sampled in this study and in many cases, more than one lineage was circulating (Table 2; Figure 4 and Figures S1–S3).

EV outbreaks have been shown to be modulated by both circulation of EVs alongside the increase in the proportion (above a threshold) of the population (impacted by both birth and death rates) that is immunologically naïve [28]. Hence, the reduction in EV detection by case-based surveillance (CBS) could be accounted for in either of three ways. It is possible, that the SARS-CoV-2 pandemic resulted in testing bias towards SARS-CoV-2 and consequently resulted in other viruses being missed especially in cases of co-infection with SARS-CoV-2 [29,30]. Secondly, the pandemic could have resulted in changes in clinic visits by patients resulting in cases not logged at hospitals [31]. Thirdly, non-pharmaceutical interventions might have prevented the proper mingling of EVs and the immunologically naïve to cause outbreaks of the expected size. If either of the first two scenarios are true, then cases might have existed but were missed by CBS. However, if the third scenario is true, the reduced EV detection by CBS in 2020 might be an indication that removing non-pharmaceutical interventions might restore circulation of EVs to pre-intervention levels. Coupled with a now larger (than in 2020) population of the immunologically naïve, it is possible that this will result in (subsequent years) a future EV outbreak that is larger than expected.

The SSW was only able to unambiguously delineate the Enterovirus C members present in the population. Like all other cell culture independent Pan-EV molecular assays that have been used to investigate EV diversity in wastewater [10,11,12,13], the assays coupled together in the SSW have been independently shown to detect members of Enterovirus A-D [11,12,32]. In fact, we recently showed that the same assay detected (in wastewater) a C1-like EV-A71 (a member of Enterovirus A species) that had been circulating silently for over two years [33]. Hence, we are confident the Enterovirus C species members detected using the SSW is not a consequence of bias from the RT-nested PCR assay (Figure 1B). Furthermore, it is important to note that while the SSW might not have resolved EV mixtures when present (Table 2), it was able to signal the possible presence of EVs (~350 bp amplicon in gels), and more importantly, the possibility that multiple EV types might be present in a single sample (multiple peaks, Table 2). In settings where access to high-throughput sequencing capacity might be scarce or unavailable, the SSW can be quite valuable. In such instances (depending on the capacity of the facility), suspected cases of EV mixtures can be resolved by cloning of amplicons prior to Sanger sequencing. Alternatively, amplicons from assay 1 (Figure 1A) can be serially diluted to the minimum concentration at which amplicons can be recovered using the SSW. Multiple aliquots of this minimum dilution (or the penultimate) can then be subjected to the SSW and all amplicons sequenced by the Sanger sequencing method.

The assays used in the LRISW (assays 3a and 3b) in this study are a blend of both assays one and two (Figure 1B, Table 1). The LRISW unambiguously detected members of three (A–C) different EV species suggesting that although the primers were paired differently (Table 1) than previously described [11,12,19,20], they retained the capacity to detect EVs across varying species. While it is impossible to say categorically that no EV genome present in the samples could have been missed, we were careful to ensure that our choice of primers and assay conditions for assay 3 were promiscuous enough to ensure that any amplicon generated in assay 1 should be further amplified in assay 3. Specifically, we selected outer primers MM-EV-F2 and CRE-R for assay 3 because we noted (as shown in Figure S6a) that binding sites for MM-EV-F2 should be present in any amplicon generated using 5-UTR and binding sites for CRE-R should be present in any amplicon generated using MM-EV-R1. To accommodate the internal variation between CRE-R and MM-EV-R1, we used a low annealing temperature (45 °C) for the first five cycles of assay 3 (Table 1). To explore the possibility that our internal primers (AN89 and AN88) might have missed some EV types, we checked the primer binding sites for AN89 and AN88 in the 3a and 3b contigs (respectively) recovered in this study (Figure S6b,c). Our findings show congruence between the specificity domain of the primer sequence [20] and their respective binding sites in the contigs (Figure S6b,c). Hence, considering the above stated and congruence between EV detection by the SSW and LRISW approaches (Table 2), we are confident the preponderance of Enterovirus C species members detected in the LRI workflow (and overall, in this study) is not a consequence of bias from the way the primers were paired (Figure 1 and Table 1). Rather, our findings show that the LRISW increased the resolution of the Pan-EV assays detecting two (A and B) more EV species and four (CVA4, EV-A76, EV-A90 and E14) more EV types than the SSW (Table 2). On the other hand, the Enterovirus C preponderance detected in this study might be a true reflection of EV dynamics in the region sampled during the 2020 EV season. Though Enterovirus C presence has been documented in USA [10], it has been suggested that this species is not as common in temperate climates as they are in the tropics [34,35]. Little attention has, however, been paid to their dynamics in Southwest US which includes desert climates (such as the region sampled in this study) and it seems, according to our findings, that Enterovirus Cs might circulate well in such.

The LRISW also increased the number and diversity of EV types per sample, enabling the detection of multiple lineages of individual EVs (Table 2 and Table S2, Figure 4 and Figures S1–S3). Specifically, the molecular clock of EVs (~1% divergence per year, [36]) suggests that the amount of divergence detected per EV type per sample (and across the sampling period) in this study (Table 2 and Table S2), can only be an indication of the presence of multiple lineages in the samples and by extension, in the population. This we confirmed by phylogenetic analysis (Figure 4 and Figures S1–S3).

It is, however, interesting to mention that when subjected to a BLASTn search of the GenBank database [26], most of the EV variants detected in this study were >4% divergent from the most similar sequence in the database but >10% divergent from the most similar sequence in the database from the USA. This suggests that the complete or near complete capsid sequence of most of the EV types circulating in the USA and detected in this study might not have been documented (or documented but not yet publicly available) for over a decade. This is not surprising as most EV surveillance studies (besides recent studies focused on EVD68 and EVA71) have leaned heavily on assays that amplify only a very small portion of EV capsid genomic region [20,37]. For example, while a GenBank search using the query ‘coxsackievirus A1′, ‘coxsackievirus A1 VP1′ and ‘coxsackievirus A1 complete genome’ yielded 82, 59 and 3 entries, respectively, a BLASTN search of the database using LRISW assay 3a (Figure 1A) contigs yielded only 10 (nine CVA1 and one CVA19) entries with >50% ‘query cover’. This dearth of complete genome or at least complete capsid sequence can be detrimental to the development of broadly acting countermeasures (BACMs) against EV capsids as it limits the amount of sequence data available for analysis and by extension both our understanding of the evolutionary dynamics of EV capsids and the identification of potential targets in EV capsids for the development of BACMs.

In addition to increasing the amount of near complete or complete EV capsid sequence data available, the LRISW (Figure 1) described here further helps our understanding of the evolutionary dynamics of EV capsid sequences by enabling the detection of linked mutations or amino acid substitutions (i.e., co-evolving sites/epistasis) in EV capsid sequences. While there are very robust bioinformatic pipelines that might enable us to predict, the likelihood that two variants detected using high-throughput sequencing strategies are linked, long-read sequencing strategies, such as LRISW, helps us detect linked mutations without the need for probabilistic predictions and consequently enables us to not only better understand co-evolution of sites along EV genomes but also the co-evolution of amino acid residues in EV capsids and beyond. This can help us better understand quasi-species dynamics in EVs which can guide us in the design, development and use of countermeasures such as vaccines and chemotherapeutic agents in the control of EVs [38].

The enterovirus members detected in this study have been previously documented in different clinical manifestations including encephalitis, paralysis or myelitis, meningitis, upper and lower respiratory disease, conjunctivitis, pleurodynia, herpangina, gastroenteritis and type 1 diabetes among others [5]. Particularly, preponderance of Enterovirus C was detected and our data suggest that recombination (which is as a result of co-infection of hosts with multiple EV types) between multiple Enterovirus C types might be occurring in the population (Figure 5, Figure 6 and Figure S5). It is important to mention that some Enterovirus C members detected in this study (CVA11 and CVA13) are known for their ability to function as recombination partners for vaccine strains of the polioviruses, resulting in the emergence of circulating vaccine-derived polioviruses (cVDPVs) in which both transmissibility and pathogenicity have been restored to wild-type poliovirus levels [39,40,41]. Though wild poliovirus seems to be currently restricted to some parts of southeast Asia [42], cVDPVs have a larger international footprint [42,43]. The fact that inactivated polio vaccine (which elicits great humoral immunity but poor gut immunity) forms the mainstay of polio vaccination in USA means introduction of live vaccine poliovirus strains by a vaccinee or its contact into the population might not cause clinical manifestation. However, the population might lack the mucosal immunity to interrupt transmission of the virus should transmission be established as documented in Israel [9,44]. With circulation in the population of multiple types and intra-type lineages of Enterovirus C members that can function as recombination partners for vaccine strains of the polioviruses, cVDPVs can emerge (as was documented in Minnesota [45]), circulate silently, and serve as a reservoir to seed other parts of the world with lower population humoral immunity to the virus. It is therefore important we monitor the types, dynamics, and distribution of Enterovirus C species members in the population.

It is important to note that all the EV variants described in this study were recovered from solids trapped in membrane filters meant to be discarded as part of the wastewater clarification protocol for concentration and detection of SARS-CoV-2 in wastewater (reviewed in [46]). Considering, it had been shown [47] that only approximately 6% of naked enteric viruses adsorb to wastewater solids (as opposed to over 20% for enveloped viruses), and this solids fraction is what we analyzed in this study, it is likely that the results of this study might only be assessing <10% of EVs in the wastewater samples screened. While the EV types detected and described here are a subset of those present in the samples screened, it is not clear how representative they are of the diversity in the samples. However, if they are representative of EV diversity in the samples, then our data might suggest a decrease in diversity from June to October 2020 in the population sampled.

Ultimately, in this study, EVs were unambiguously detected in 20 of 48 samples screened. It should however be noted that inability to detect any virus of interest in an environmental sample such as that described here might not necessarily mean absence of the virus of interest in the sample. Non-detects could be due to the presence of PCR inhibitors in some samples, fragmented viral genome, very low concentration of genome of interest in sample, non-specific off target amplification (due to the diversity of nucleic acid types in such matrices), primer mismatch among others. Some of the above mentioned might even account for the unresolved multiple peaks, discrepancies in EV types recovered at ST1-d105 and ST5-d28, and discrepancies in contig recovery for by assays 3a and 3b of the LRISW (Table 2).

We acknowledge that a more sensitive approach, coupling screening of both solids trapped in filters and concentrates made using centrifugal filters or polyethylene glycol (which potentially contain the remaining 94% of naked viruses) with the pan-EV amplicon-based long-read high-throughput sequencing strategy described here, will definitely be more revealing in describing EV dynamics in the population and might better enlighten on the meaning of the Enterovirus C abundance found here. Conversely, if sampling <10% of EV diversity in wastewater in this population is this informative, questions remain about how much virus diversity (especially with regard to variants of interest, concern or high consequence) is lost by performing the clarification step in the WW concentration protocol for SARS-CoV-2. It might therefore be necessary to account for the amount of SARS-CoV-2 (and other enveloped viruses) genetic material we might be losing to this filtration step. This is also significant for samples we classify as ‘negative’ or ‘below detection limit’.

## Figures and Tables

**Table 1:** Variation in assay conditions for the PCR assays used in this study. All primers listed have the same sequence as those listed in the references cited in the table. Please see Figure S6 for primer sequences. The LG prefix in primers for assays 3a and 3b implies the Loop Genomics adapter was added to the 5′-end of these primers.

Assay Name	Primer Name	Annealing Temperatures	Extension Temperature and Time	Amplicon Size (bp)	Reference
1a	5-UTR	55 °C for 30 s, 32×	68 °C for 8 min	~3900	[19]
	CRE-R				
1b	MM-EV-F2	55 °C for 30 s, 32×	68 °C for 8 min	~3900	[12]
	MM-EV-R1				
2	AN89	45 °C for 30 s, 5× 60 °C for 30 s, 30×	72 °C for 30 s	~350	[20]
	AN88				
3a	LG-MM-EV-F2	45 °C for 30 s, 5× 55 °C for 30 s, 35×	68 °C for 3 min	~2400	This study
	LG-AN88				
3b	LG-AN89	45 °C for 30 s, 5× 55 °C for 30 s, 35×	68 °C for 3 min	~1900	This study
	LG-CRE-R				

**Table 2:** Details of EV types recovered using the Sanger and LRI workflows.

		SSW	LRISW			
Site #	Sampling Day	EV Type	Assay 3a EV Type (#)	Assay 3a Divergence (%)	Assay 3b EV Type (#)	Assay 3b Divergence (%)
1	105	CVA13	EV-A90 (2)	0.64		
2	70	Multiple peaks—EV type undetermined				
3	0	Multiple peaks—EV type undetermined	EV-A76 (8)	0.18–0.55	EV-A76 (14)	0.11–0.54
			E14 (1)	NA	E14 (8)	0.05–0.58
			CVA19 (6)	0.05–0.41		
	105	CVA19	CVA19 (20)	0.05–0.91		
4	0	Multiple peaks—EV type undetermined	EV-A76 (2)	0.32	EV-A76 (7)	0.05–0.48
			CVA1 (6)	0.36–16.63	CVA1 (19)	0.05–16.70
			CVA19 (2)	0.23	CVA19 (3)	1.07–1.35
					CVA13 (9)	0.05–0.59
	70	CVA19	CVA19 (2)	0.36		
	105	CVA19	CVA19 (52)	0.09–24.37		
5	0	CVA24	CVA24 (23)	0.04–1.31	CVA24 (80)	0.05–0.96
			CVA4 (2)	0.65	CVA4 (13)	0.05–0.48
	28	CVA24				
	70	Multiple peaks—EV type undetermined				
	105	CVA1	CVA1 (4)	0.23–0.50		
6	28	Multiple peaks—EV type undetermined				
7	0	Multiple peaks—EV type undetermined	EVA76 (12)	0.18–0.60	EVA76 (15)	0.11–0.54
			CVA1(21)	0.05–5.97	CVA1 (25)	0.05–4.48
			CVA13 (1)	NA	CVA13 (15)	0.05–28.08
			CVA11(2)	15.17		
			CVA19 (9)	0.27–0.73		
	28	Multiple peaks—EV type undetermined				
	105	CVA19	CVA19 (16)	0.27–24.63		
8	70	CVA13	CVA13 (10)	0.09–0.72	CVA13 (2)	0.05
9	0	Multiple peaks—EV type undetermined	CVA1(39)	0.05–14.97	CVA1 (92)	0.05–10.81
			CVA19 (33)	0.05–5.66	CVA19 (19)	0.16–2.79
	28	Multiple peaks—EV type undetermined				
	105	CVA19	CVA19 (20)	0.14–0.82		
10	0	CVA11	CVA11 (18)	0.09–0.63	CVA11 (65)	0.05–0.64
	105	CVA11	CVA11 (2)	0.72	CVA11 (1)	NA
11	0	CVA13	CVA13 (74)	0.04–0.67	CVA13 (135)	0.05–0.85
	105	CVA1	CVA19 (2)	0.41		
					CVA1 (1)	NA
12	70	CVA19	CVA19 (2)	0.59	CVA19 (1)	NA
13	70	CVA11	CVA11 (2)	0.40	CVA11 (1)	NA
	**Total Variants**	16	393		525	

NA—not applicable. Black = congruence of EV type by SSW and LRISW. Purple = congruence of EV type by SSW and LRISW but more EVs detected by LRISW than SSW. Red = multiple peaks by SSW but resolved by LRISW. Orange= multiple peaks by SSW and unresolved by LRISW. Green = discordance between EV type by SSW and LRISW. # means Number of variants detected.

**Table 3:** Range of number of small reads that mapped to each of the EV LRISW contigs in Table 2 (and Table S1)—EV long-read contigs (column 6) and the corresponding range of contig coverage. Please note that the S/N column was added for clarity only, to show the number of samples (a serial count of 1–19) from which reads were recovered using LRISW (see Section 3.2.2) (i.e., there are 19 samples ST1-d105 to ST13-d70 from which reads were recovered). Hence, all data described here are for the LRISW. # means Number.

			Assay 3a		Assay 3b	
S/N	Site No.	Sampling Day	Highest # of Small Reads/Contig	Smallest # of Small Reads/Contig	Highest # of Small Reads/Contig	Smallest # of Small Reads/Contig
1	1	105	1740	1234	NA	NA
2	3	0	1528	252	1950	406
3	3	105	1754	244	NA	NA
4	4	0	1566	356	2028	196
5	4	70	828	788	NA	NA
6	4	105	1878	230	NA	NA
7	5	0	1710	368	2258	198
8	5	105	1280	364	NA	NA
9	7	0	1776	202	2332	152
10	7	105	1724	256	NA	NA
11	8	70	1518	178	1056	476
12	9	0	1884	240	2142	124
13	9	105	1785	342	NA	NA
14	10	0	1626	264	2108	100
15	10	105	1506	484	1488	1488
16	11	0	1880	150	2240	150
17	11	105	864	168	1637	1637
18	12	70	1162	260	842	700
19	13	70	834	400	958	958
		Range # of small reads/contigs	1884	150	2332	100
		Coverage range	235.5×	18.75×	368.21×	15.79×

## Data Availability

Sequences generated from this study are available in NCBI GenBank under accession numbers MW817168-MW817183, MZ561707-MZ562246 and MZ579712-MZ580089.

## Supplementary Materials

The Supplementary Materials are available online at https://www.mdpi.com/article/10.3390/v13091803/s1. Reference [48] is cited in the Supplementary Materials.
