# Supplementary material for: Wastewater-Based Epidemiology and Long-Read Sequencing to Identify Enterovirus Circulation in Three Municipalities in Maricopa County, Arizona, Southwest United States between June and October 2020
Source: Viruses. 2021 Sep 10;13(9):1803. doi: 10.3390/v13091803 (PMC8472758; doi:10.3390/v13091803)
Supplement: Supplementary file 1 [file viruses-13-01803-s001.zip › viruses-1330803-supplementary.pdf]

Table S1. Sequencing details for each site and sampling day including reads, contigs, and variants of EV types recovered using the Sanger and LRI workflows.

		SSW	LRISW							
**Site #**	**Sampling day**	**EV-type**	**Raw Reads**	**Total Contigs**	**EV contigs**	**Deduplicated EV contigs**	**Assay 3a variants (~2,400bp)**	**Assay 3a EV-type (#)**	**Assay 3b variants (~1,900bp)**	**Assay 3b EV-type (#)**
1	105	CVA13	812,910	4281	2	2	2	EV-A90 (2)		
2	70	Multiple Peaks EV Type Undetermined								
3	0	Multiple Peaks EV Type Undetermined	3,471,817	6892	51	37	15	EV-A76 (8)	22	EV-A76 (14)
								E14 (1)		E14 (8)
								CVA19 (6)		
	105	CVA19	1,290,115	2895	31	20	20	CVA19 (20)		
4	0	Multiple Peaks EV Type Undetermined	866,354	1637	65	48	10	EV-A76 (2)	38	EV-A76 (7)
								CVA1 (6)		CVA1 (19)
								CVA19 (2)		CVA19 (3)
										CVA13 (9)
	70	CVA19	1,589,616	3947	8	2	2	CVA19 (2)		
	105	CVA19	1,016,499	2941	72	52	52	CVA19 (52)		
5	0	CVA24	2,980,818	4743	175	118	25	CVA24 (23)	93	CVA24 (80)
								CVA4 (2)		CVA4 (13)
	28	CVA24	395,904	1081	0					

	70	Multiple Peaks EV Type Undetermined								
	105	CVA1	1,489,974	2625	6	4	4	CVA1 (4)		
6	28	Multiple Peaks EV Type Undetermined								
7	0	Multiple Peaks EV Type Undetermined	1,640,050	2853	130	100	45	EVA76 (12)	55	EVA76 (15)
								CVA1(21)		CVA1 (25)
								CVA13 (1)		CVA13 (15)
								CVA11(2)		
								CVA19 (9)		
	28	Multiple Peaks EV Type Undetermined								
	105	CVA19	714,114	1929	16	16	16	CVA19 (16)		
8	70	CVA13	1,378,413	3917	15	12	10	CVA13 (10)	2	CVA13 (2)
9	0	Multiple Peaks EV Type Undetermined	1,498,698	2384	240	183	72	CVA1(39)	111	CVA1 (92)
								CVA19 (33)		CVA19 (19)
	28	Multiple Peaks EV Type Undetermined								
	105	CVA19	555,962	3305	24	20	20	CVA19 (20)		
10	0	CVA11	668,821	808	113	83	18	CVA11 (18)	65	CVA11 (65)
	105	CVA11	1,893,039	4164	4	3	2	CVA11 (2)	1	CVA11 (1)
11	0	CVA13	1,864,462	4499	273	209	74	CVA13 (74)	135	CVA13 (135)
	105	CVA1	119,868	749	6	2	2	CVA19 (2)		
						1			1	CVA1 (1)

12	70	CVA19	565,365	3355	7	3	2	CVA19 (2)	1	CVA19 (1)
13	70	CVA11	574,410	2192	5	3	2	CVA11 (2)	1	CVA11 (1)
	**Total**		**25,387,209**	**61,197**	**1,243**	**918**	**393**		**525**	

Table S2. Divergence per EV type and region recovered using LRIS workflow. # means *Number of*

			Assay 3a				Assay 3b			
**S/N**	**EV-Species**	**EV-Type**	**Maximum divergence (%)**	**# variants**	**# positive Samples**	**# days with positive samples (days)**	**Maximum divergence (%)**	**# variants**	**# positive samples**	**# days with positive samples (days)**
1	EV-A	CVA4	0.65	2	1	1	0.48	13	1	1
2	EV-A	EV-A76	11.53	22	3	1	9.18	36	3	1
3	EV-A	EV-A90	0.64	2	1	1	NA	0	NA	0
4	EV-B	E14	NA	1	1	1	0.58	8	1	1
5	EV-C	CVA1	26.16	70	4	2 (0,105)	20.18	137	5	2 (0,105)
6	EV-C	CVA11	17.68	24	4	3 (0,70,105)	2.32	67	3	3 (0,70,105)
7	EV-C	CVA13	3.73	85	3	2 (0,70)	28.08	161	4	2 (0,70)
8	EV-C	CVA19	25.01	164	11	3 (0,70,105)	15.96	23	3	2 (0,70)
9	EV-C	CVA24	1.31	23	1	1	0.96	80	1	1

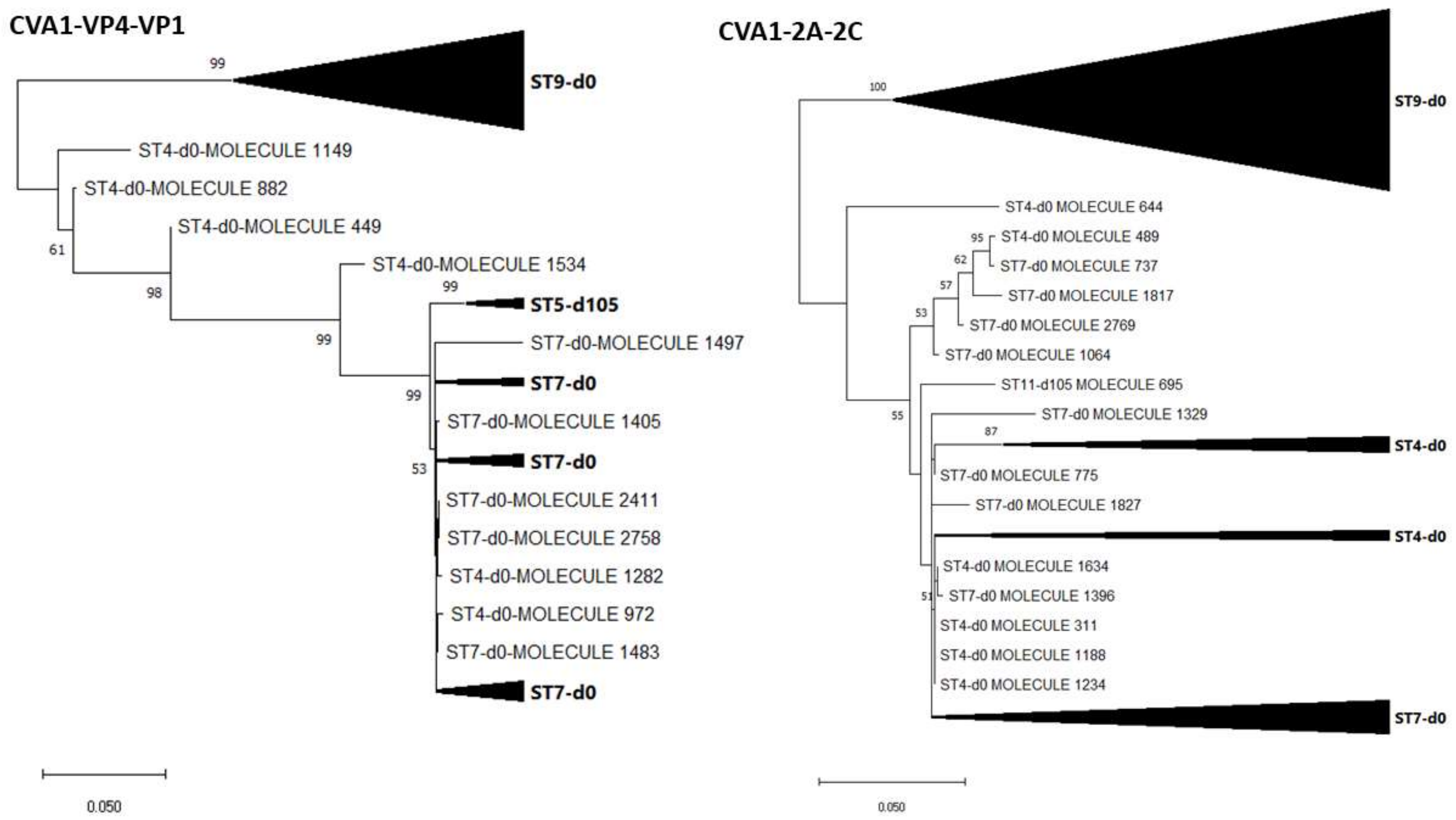

Figure S1: Maximum likelihood phylogenetic tree of CVA1 VP4-VP1 and 2A-2C contigs recovered from assay 3 (LRISW) in this study. Bootstrap support is shown if >50. Abbreviation: ST = site. We collapsed taxa for all contigs that belong to the same day and site.

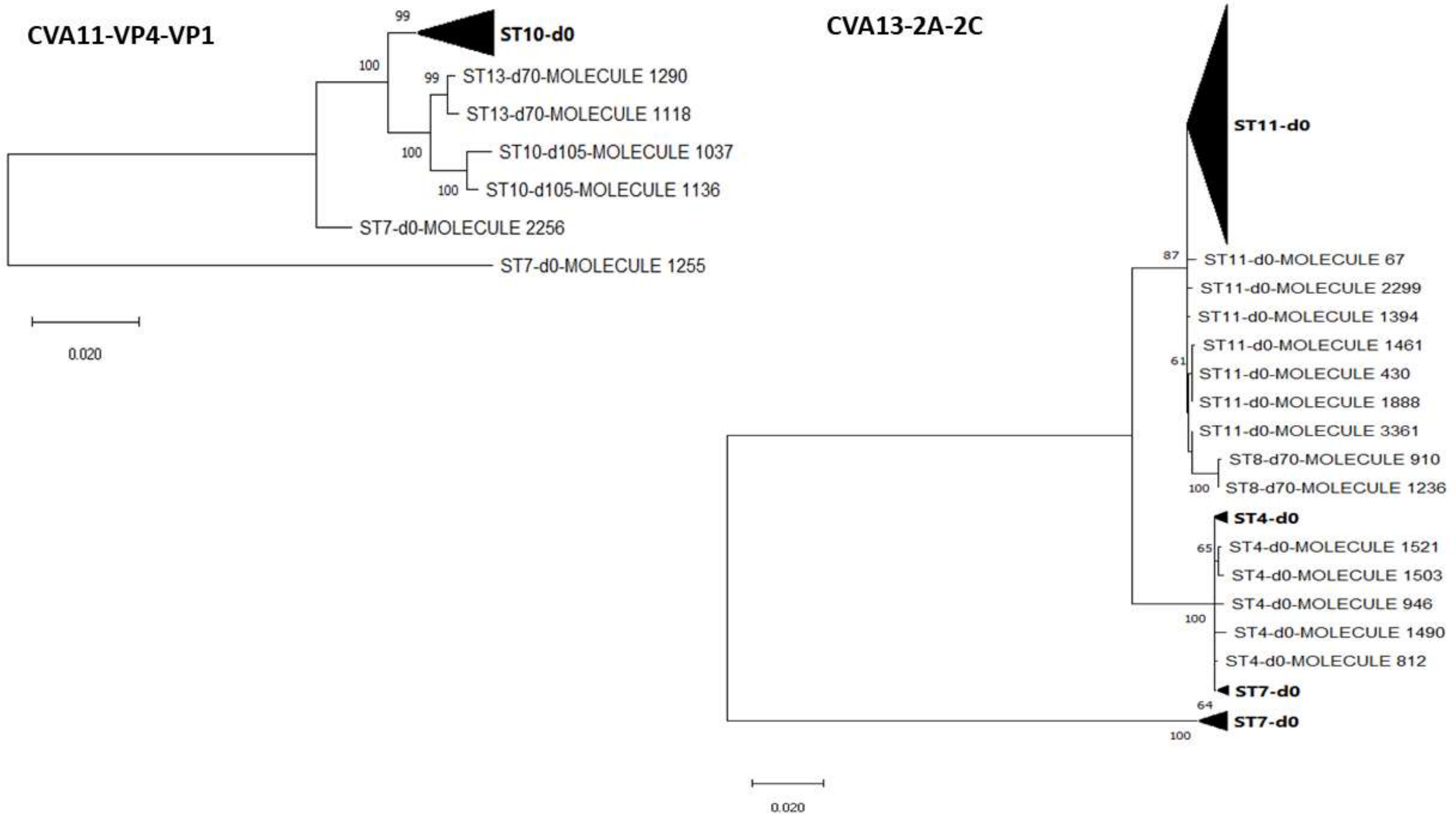

Figure S2: Maximum likelihood phylogenetic tree of CVA11 VP4-VP1 and CVA13 2A-2C contigs recovered from assay 3 (LRISW) in this study. Bootstrap support is shown if >50. Abbreviation: ST = site. We collapsed taxa for all contigs that belong to the same day and site.

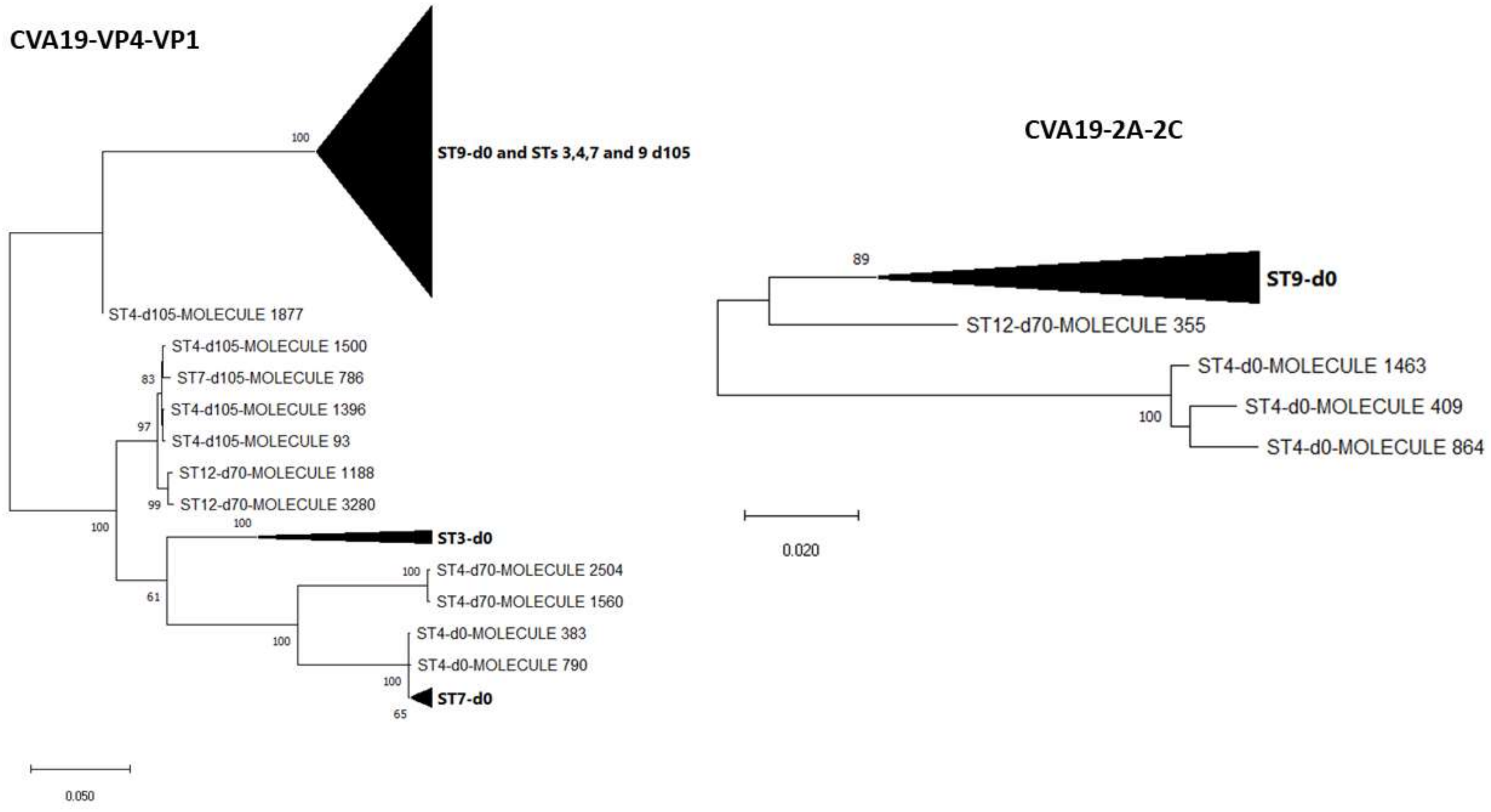

Figure S3: Maximum likelihood phylogenetic tree of CVA19 VP4-VP1 and 2A-2C contigs recovered from assay 3 (LRISW) in this study. Bootstrap support is shown if >50. Abbreviation: ST = site. We collapsed taxa for all contigs that belong to the same day and site.

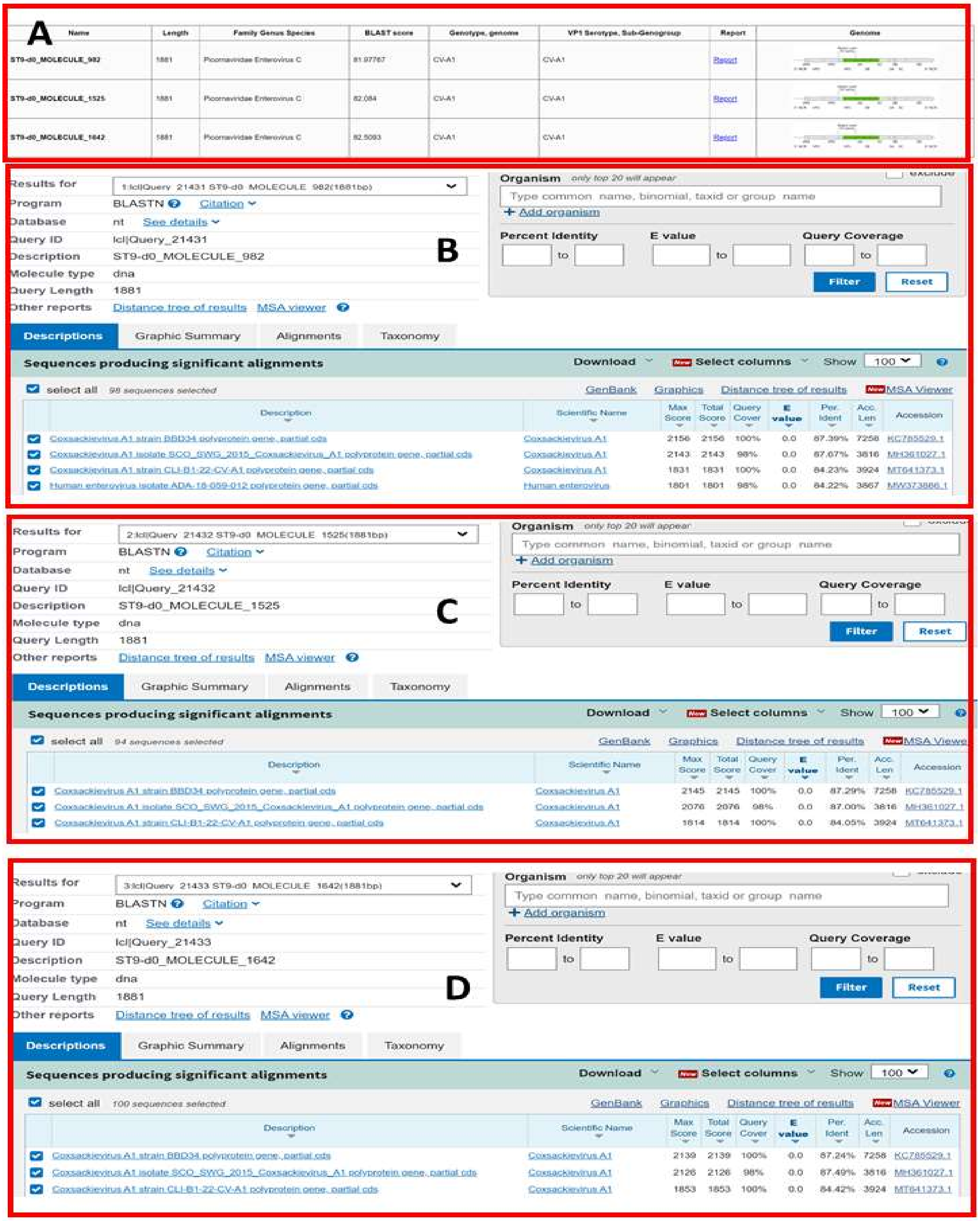

Figure S4: Repeat identification of CVA1 contigs 982, 1525 and 1642. A) EGT identification result. B-D) BLASTn results.

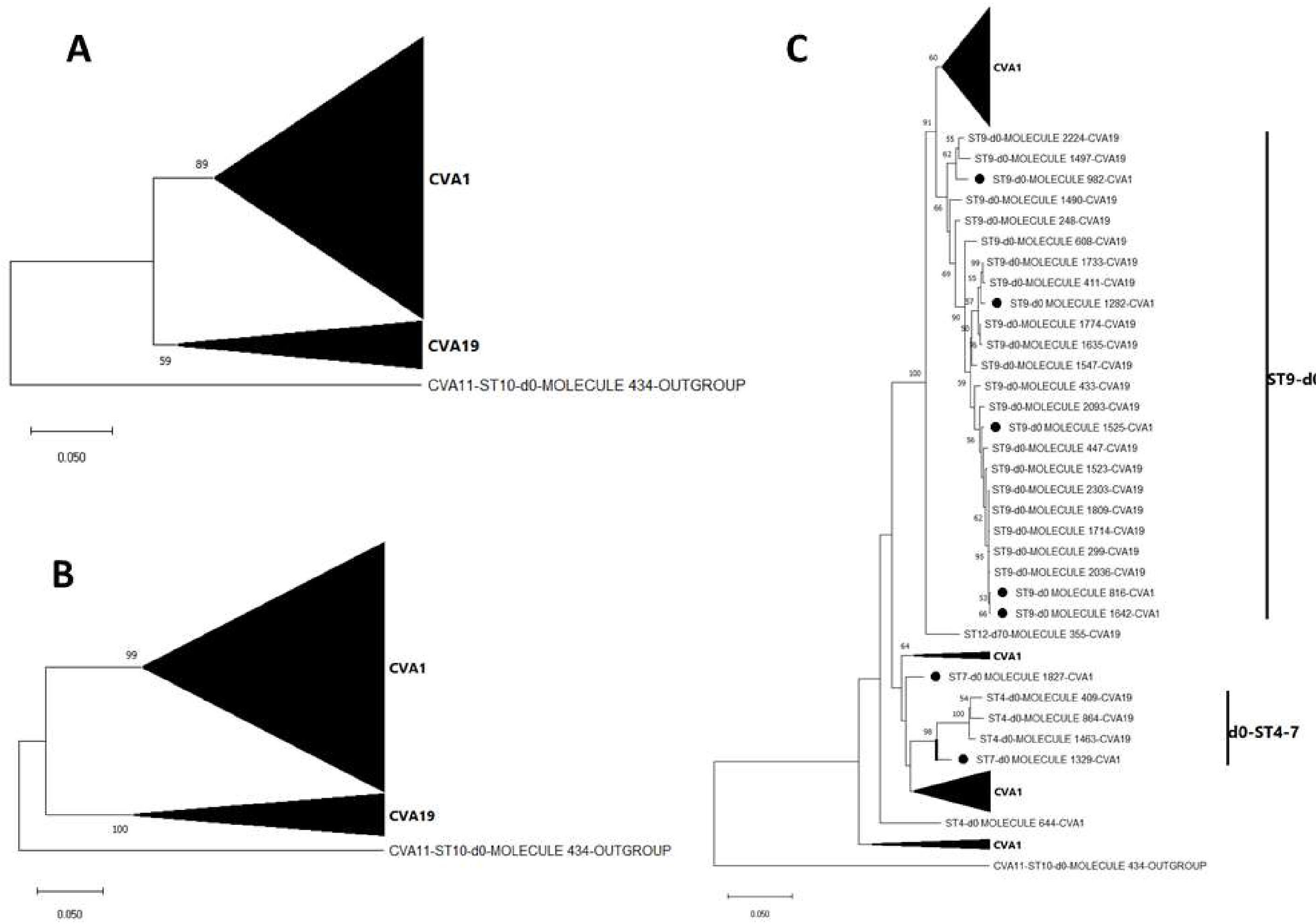

Figure S5: Neighbor-Joining phylogenetic tree of CVA1 and CVA19 contigs recovered from assay 3b (LRISW) in this study (A) VP1-2C (B) VP1 only and (C) 2A-2C. In figure S5, the CVA1 contigs that do not cluster with other CVA1s are indicated with black circles. Bootstrap support is shown if >50. Abbreviation: ST = site. We collapsed taxa for all contigs that belong to the same day and site.

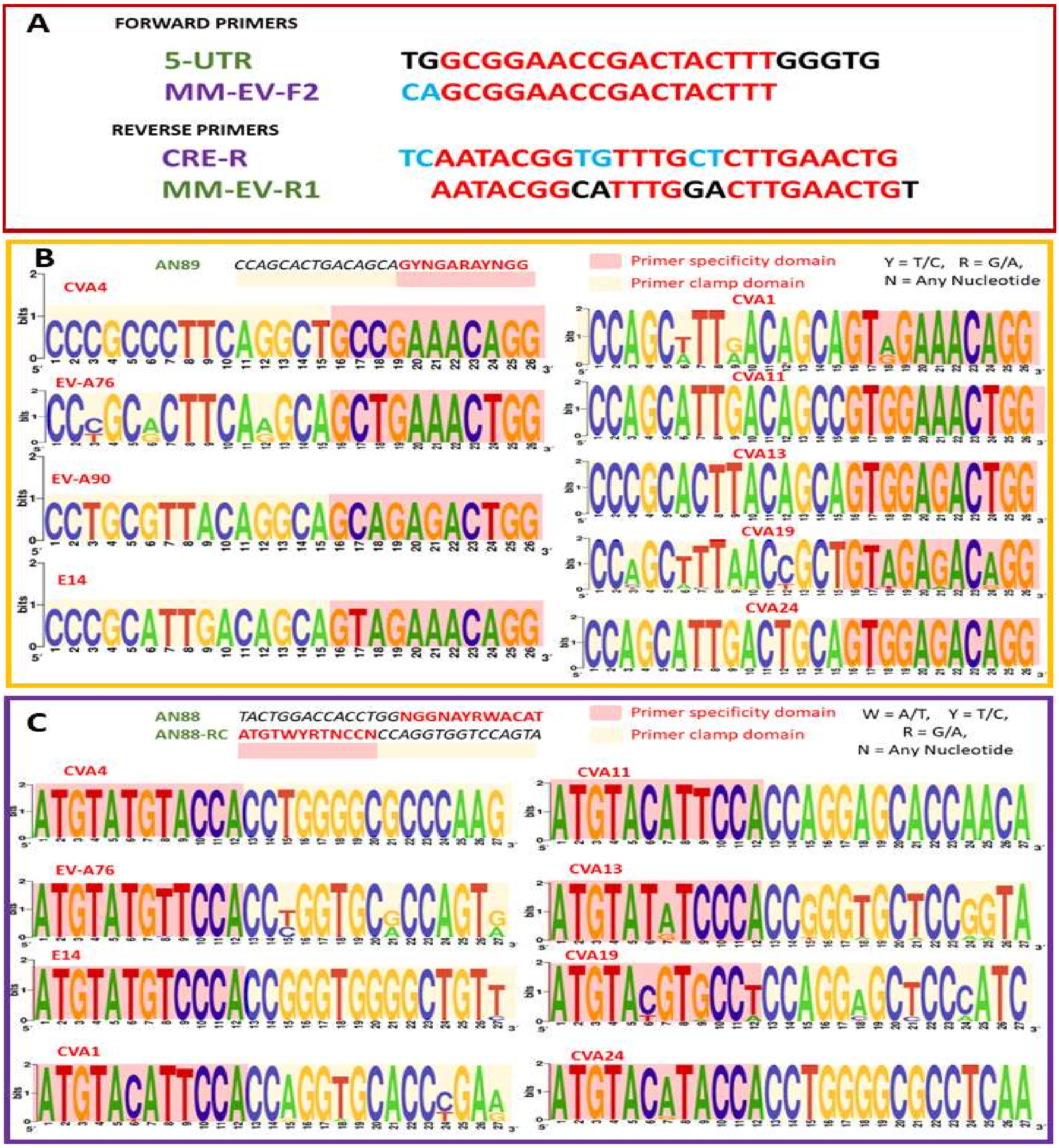

Figure S6: (A) Alignment of forward (5-UTR and MM-EV-F2) and reverse (CRE-R and MM-EV-R1) primers used in assay 1a (5-UTR + CRE-R) and 1b (MM-EV-F2 + MM-EV-R1). (B and C) The primer binding sites for AN89 (figure 1b) and AN88 (figure 1c) in all contigs generated in this study. All contigs were aligned by EV type and the primer binding sites were extracted. WebLogo online tool [1] was then used to create a representation of the consensus sequence highlighting variations when present. Primer specificity and clamp domains as described in Nix et al., 2006 are highlighted in pink and yellow, respectively. AN88-RC (figure S1C) means the reverse complement of primer AN88.

Reference

1. Weblogo available online at https://weblogo.berkeley.edu/logo.cgi (accessed on August 30, 2021)
